# Performance enhancement of short-term wind speed forecasting model using Realtime data

**DOI:** 10.1371/journal.pone.0302664

**Published:** 2024-05-31

**Authors:** Maria Ashraf, Bushra Raza, Maryam Arshad, Bilal Muhammad Khan, Syed Sajjad Haider Zaidi

**Affiliations:** Department of Electronic and Power Engineering, National University of Sciences and Technology, Karachi, Pakistan; Air University, PAKISTAN

## Abstract

The ever-increasing demand for electricity has presented a grave threat to traditional energy sources, which are finite, rapidly depleting, and have a detrimental environmental impact. These shortcomings of conventional energy resources have caused the globe to switch from traditional to renewable energy sources. Wind power significantly contributes to carbon-free energy because it is widely accessible, inexpensive, and produces no harmful emissions. Better and more efficient renewable wind power production relies on accurate wind speed predictions. Accurate short-term wind speed forecasting is essential for effectively handling unsteady wind power generation and ensuring that wind turbines operate safely. The significant stochastic nature of the wind speed and its dynamic unpredictability makes it difficult to forecast. This paper develops a hybrid model, L-LG-S, for precise short-term wind speed forecasting to address problems in wind speed forecasting. In this research, state-of-the-art machine learning and deep learning algorithms employed in wind speed forecasting are compared with the proposed approach. The effectiveness of the proposed hybrid model is tested using real-world wind speed data from a wind turbine located in the city of Karachi, Pakistan. Moreover, the mean square error (MSE), root mean square error (RMSE), and mean absolute error (MAE) are used as accuracy evaluation indices. Experimental results show that the proposed model outperforms the state-of-the-art legacy models in terms of accuracy for short-term wind speed in training, validation and test predictions by 98% respectively.

## Ⅰ. Introduction

**Fig 1:**
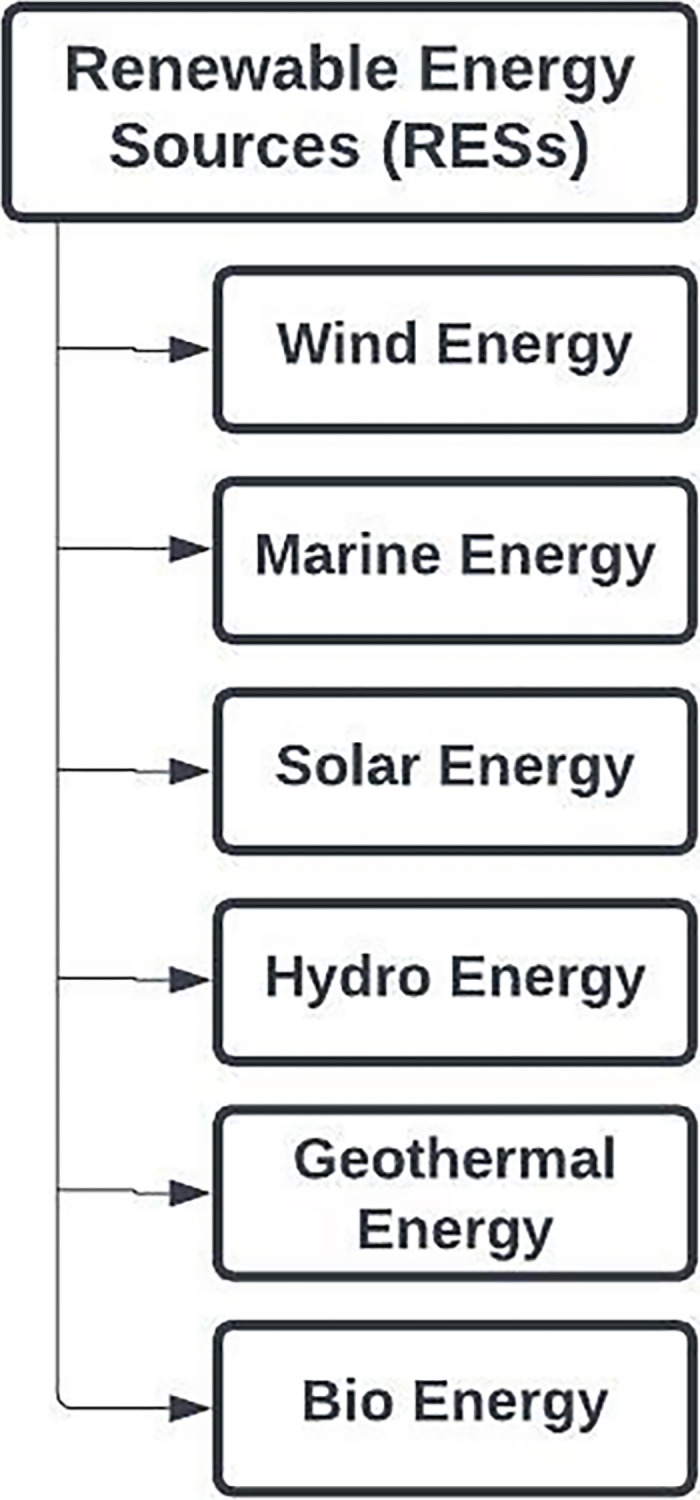
Types of renewable energy sources (RESs).

**Fig 2:**
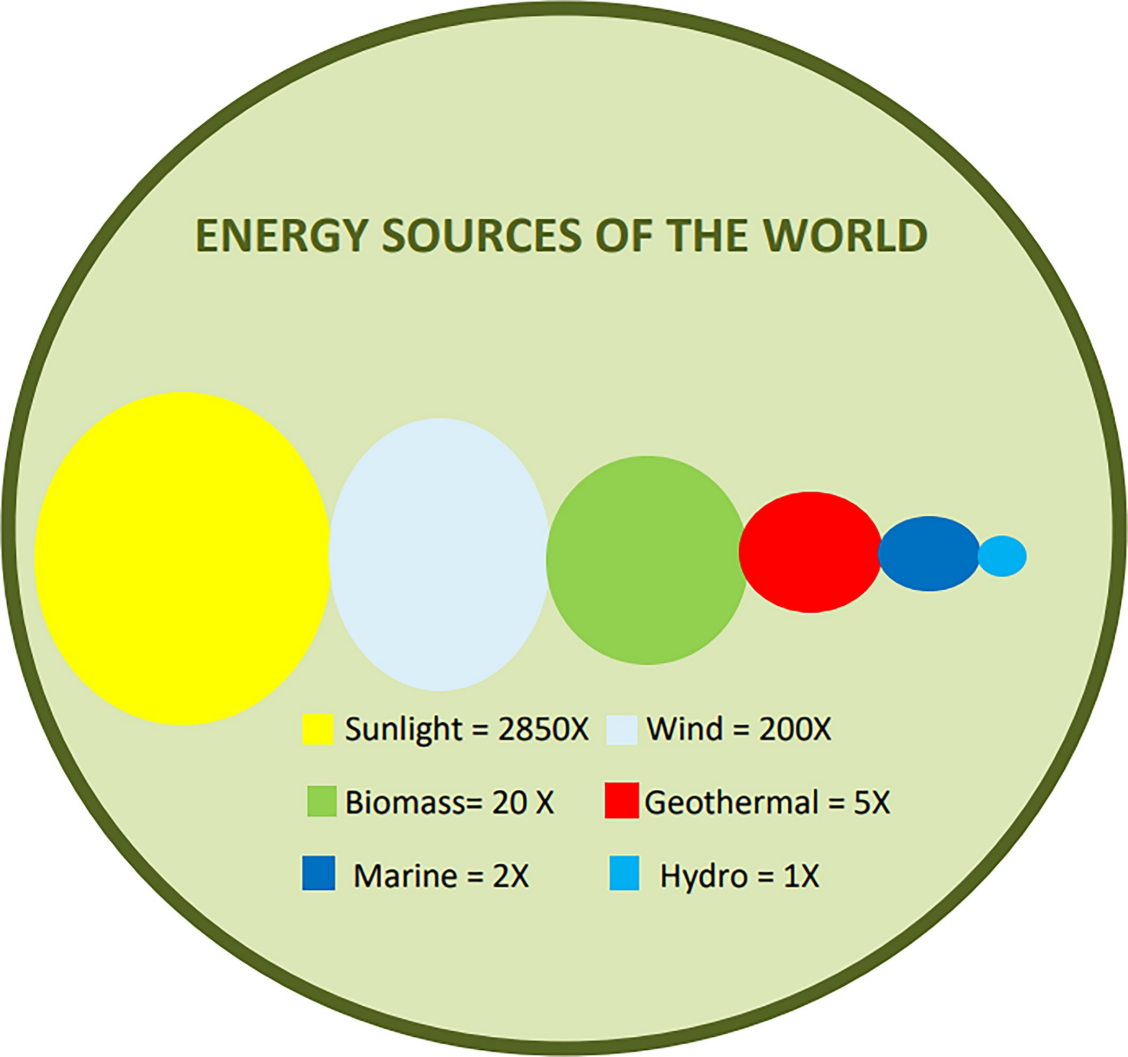
Energy sources of the world [6].

Energy demand is rising globally, especially regarding electrical energy, along with the escalating effects of problems like the decreasing supply of fossil fuels and their adverse impact on the environment, rising oil prices, and their combined products on civilizations [1, 2]. To meet global energy demand and reduce greenhouse gas emissions according to the Paris Agreement [3], the globe is undergoing a switch from fossil fuels to renewable energy sources (RESs). RESs are defined as clean forms of energy as they produce zero carbon emissions [4]. All energy that comes from natural movements and mechanisms of the environment is considered renewable energy. Renewable energy excludes fossil fuels, waste from fossil reserves, and trash from inorganic resources [5]. Fig 1 illustrates renewable energy supply types. RESs convert this natural source of energy into useful forms of energy like electricity. Fig 2 depicts the capacity of renewable energy sources to supply more than 3000 times the present global energy demand [6]. Among RESs, wind power is gaining attention considering its future potential to meet growing electricity demands at a reasonable price. In 2022, 50% of the world’s consumption of green energy came from wind, which grew at a rate of 13.5%, while 31.43% came from solar [7]. However, wind power production relies highly on wind speed, which is stochastic and fluctuating. For this reason, it is crucial to forecast wind speed accurately for the safe operation of wind turbines and the integration of wind power with the grid.

Machine learning is an emerging area and currently many applications are being targeted in order to solve practical problems ranging from Driver’s Diabetes Monitoring System, Vehicular Ad-Hoc Networks, Task offloading optimization and energy management system, and Offload micro services based applications [8–11]. However, in this paper we are implementing machine learning algorithms in wind energy domain which is an offshoot of machine learning to resolve more practical problem of wind speed forecast in wind power generation.

For predicting wind speed, several approaches have been put forth; each has its own advantages and limitations and can be used with different datasets. It is difficult for general models to faithfully represent the development patterns buried in large and heavily sampled datasets. Physical model-based approaches and statistical modelling techniques are the two main groups into which wind speed prediction procedures can be classified [12]. A set of physical models with numerical parameters that characterize local meteorological and geographic characteristics such as temperature, atmospheric pressure, surface roughness, and barriers are the foundation of numerical weather prediction (NWP) systems [13, 14]. Supercomputers are used for performing NWPs because of the massive amount of processing power required. Due to the difficulties in gaining information quickly and the related high expenses, NWPs are typically conducted once or twice daily. When the weather is stable, these models work well for long-term forecasting [15]. The statistical method leverages the difference between the estimated and actual wind speeds in the recent past to fine-tune model parameters. It is based on training with measurement data. It’s simple to model, cheap, accurate and can make predictions in real-time. It is based on patterns rather than any pre-established mathematical formula. If prints are matched with historical ones, errors are reduced. Neural network (NN) and Time-series-based models based approaches are two subcategories of this method. These methods are considered accurate for short-term and medium to long-term wind speed prediction (WSP) [16].

With the explosion of interest in data science over the past few years, statistical models have come into greater focus [17]. Machine learning (ML) methods [18], such as artificial neural networks (ANN) [19], support vector machines (SVM) [20], extreme learning machines (ELM) [21], and deep learning (DL) [22], are developing remarkably quickly. In this paper, we are exploiting the power of machine learning in short-term wind speed forecasting and propose an ensemble L-LG-S model for accurate predictions. This model uses an LSTM component to analyze historical wind speed data and identify recurrent patterns. The LightGBM component focuses on integrating features and identifies intricate connections between wind speed and other pertinent variables. By considering both linear and non-linear correlations in the data, the SVR component adjusts the predictions given by the LSTM and LightGBM models. All three components’ projections are combined to form the final forecast. The rest of the article is divided into the following sections. The literature review is presented in Section II. The related methodologies are given in Section III. The proposed model establishment is presented in Section IV. Results and analysis are presented in Section V. Section VI presents the conclusion and future work is given in Section VII.

## II. Related literature

The statistical methods are widely used in the literature for short-term wind speed prediction (WSP). Time series models such as ARIMA [23–25], ARMA [26, 27], AR [28], ARIMAX [29, 30], ARX [31], etc, have shown remarkable accuracy in predicting wind speed. However, they require high-order modeling and more computational power to capture nonlinearity and the stochastic nature of wind speed. Initially, time series models are best known for linear systems. On the contrary, with the advancement in ANN, ML, and DL techniques, they are getting more attention in the forecast area. Many researchers have used them single-handily for WSP and developed more advanced hybrid models to forecast short-term wind speed accurately. A few types of research are presented in Table 1 which shows the effectiveness of Machine learning models in wind speed forecast.

**Table 1:** Overview of literature review.

References	Research Area	Methodology	Results
[32]	Short-term wind speed prediction	UKF combined with SVR in state-space model	The proposed method excels in one-step and multi-step WSP predictions.
[33]	Wind speeds prediction at an ultra-short-term scale while considering a range of climate factors	Mathematical morphology (MM) decomposer and two LSTM networks	Ideal for non-stationary time-series forecasting
[19]	Short-term wind speed forecasting	HC-VMD-GA-BP based WSP model.	The fusion model excels in accuracy and efficiency.
[34]	Addressing gradient information vanishing or exploding during training	LSTM and deep neural networks	The proposed model significantly boosts wind speed prediction accuracy.
[35]	Handling nonlinear features within a massive dataset	Data-mining-based machine learning framework	Improved the prediction accuracy up to 28.33%
[36]	Enhancing forecast accuracy	RNN prediction and error correction methods	The hybrid system surpasses individual models and traditional approaches.
[37]	Wind speed forecast at each short-term time step	Probability-density-based wind speed prediction model	Significant improvement in wind speed prediction model performance.
[38]	To enhance predictability of wind speed	Prediction model based on GRUs (Gated Recurrent Units)	Achieved better computation speed and accuracy than SARIMAX and LSTM.
[39]	Ultra Short Term Wind Speed Prediction	Hybrid model using SARIMAX, RNN, and SVR	Model outperforms individual models in forecasting accuracy.
[40]	Prediction and evaluation of the error of various neural network model.	Prediction method based on ICEEMDAN, BPNN, CNN, RNN, and LRN.	The hybrid model outperforms single neural networks in accuracy and stability
[41]	To address the multistep wind speed prediction problem	Encoder–decoder architecture	The 3-hour multistep EEMD-Transformer model reduced MAE by 50.61% and RMSE by 56.88%
[42]	To develop novel wind speed prediction scheme	Estimation with EKF and prediction via NN extrapolation	Results reveal that with a longer forecasting horizon, predictions worsen
[43]	Novel method for short-term WSP	Jaya-SVM model	The proposed model outperform XGBoost model, MLPR model, LASSO model, DBN model, GPR model, SSAE model and GrC model
[44]	for two dimensional wind speed forecast	CNN and LSTM for improved WSP accuracy.	The model outperformed persistence, ANN, and LSTM models.

## III. Related methodologies

### Convolutional neural network algorithm

Convolutional neural networks, often called CNNs or ConvNets, were developed in the late 1990s and are a feed-forward artificial neural network whose connectivity topology is modeled after the structure of the animal visual cortex. Due to the input layer, output layer, several hidden layers, and millions of parameters that CNNs contain, they are able to learn complex objects and patterns. It pools and convolves the input to create smaller samples before applying the activation function. The output layer is the last completely linked layer after all the hidden layers, which were originally only partially connected [45, 46]. The general equation for CNN [47] is presented in Eq no 1:

y=σ(W*x+b) (1)

where,

- x represents the input image or feature map,
- W is the set of learnable convolutional filters,
- b is the bias term,
- σ is the activation function, commonly ReLU (Rectified Linear Unit),
- y represents the output feature map

### Recurrent neural network algorithm

RNN stands for a recurrent neural network. It is a neural network that processes time series, audio, and spoken language sequential data. RNNs, which analyze input data in a single pass, as opposed to feedforward neural networks, are designed to handle data sequences and can store information about earlier inputs. In sequential data, they can now spot patterns and temporal correlations [48]. A simple RNN model [49] can be described by the Eqs (2, 3):

$$h_{t}=\sigma (W_{hh}h_{t-1}+W_{xh}x_{t}+b_{h})$$ (2)

$$y_{t}=\mathrm{SOFTMAX}(W_{hy}H_{t}+B_{y})$$ (3)

where,

$$•\;h_{t}=\sigma (W_{hh}h_{t-1}+W_{xh}x_{t}+b_{h})$$

The hidden state h_*t*_ at time step *t* is computed by applying the activation function σ to the sum of the weighted previous hidden state h_*t*_, the weighted current input *x*_*t*_, and the bias vector b_*h*_.


$$•\;y_{t}=\mathrm{soft}\;\max\left(W_{hy}h_{t}+b_{y}\right)$$

The output h_*t*_ at time step *t* is computed by applying the softmax function to the sum of the weighted current hidden state h_*t*_, the weighted matrix W_*hy*_, and the bias vector b_*h*_.

### Lazyprophet algorithm

LazyProphet is a potent time series prediction approach [50]. It functions by connecting the start of the time series to a spot halfway through, and then connecting the midpoint spot to the end of the series. This procedure is repeated multiple times, and the position of the”kink” is simultaneously altered (intermediate node). The slope of each line from the midway to the final spot is further penalized by the addition of the”decay” component. Thus, this model is known as”LazyProphet” because it is less demanding.

### LightGBM algorithm

In 2017, Microsoft developed the LightGBM gradient boosting framework. The decision-tree-based algorithm emphasizes high efficiency, scalability, and prediction accuracy [51]. To make predictions, LightGBM builds an ensemble of gradient-boosting decision trees, where each tree is trained to reduce the residual error of the prior tree. The training procedure employs a leaf-wise method, where each tree is formed by recursively splitting the data at the point that leads to a significant reduction in the loss function. LightGBM additionally uses regularization, early halting, and feature subsampling to minimize overfitting. LightGBM model [52] equation is described in Eq 4:

$$F(x)=\sum_{m=1}^{M}f_{m}(x)$$ (4)

where, *f*_*m*_(*x*) is the output of the mth weak regression tree and *F*(*x*) is the ultimate result.

### LSTM algorithm

Hochreiter and Schmidhuber first introduced the Long Short-Term Memory (LSTM) technique as a sort of recurrent neural network (RNN) architecture in 1997 [53]. Traditional RNNs have a problem with vanishing gradients that can make it challenging to learn long-term dependencies in time series data. LSTMs are made to solve this issue. The input gate, forget gate, output gate, memory cell, and other components make up the LSTM architecture. The gates control the flow of information into and out of the memory cell, which is in charge of storing and updating data over time. Moreover, the LSTM architecture has optional dropout layers to avoid overfitting. The loss function, commonly mean squared error, is minimized by training the LSTM model via backpropagation through time (BPTT) (MSE). An optimization technique like Adam or RMSprop is used to iteratively change the weights and biases of the LSTM layers. Once trained, the LSTM model can be used to predict outcomes in the future. The LSTM model receives historical time series values as input and forecasts a single value for the following time step. The Eqs (5–10) describe the LSTM components [54]:

$$f_{t}=\sigma (V_{f}[h_{t-1},x_{t}]+a_{f})$$ (5)

$$i_{t}=\sigma (V_{i}[h_{t-1},x_{t}]+a_{i})$$ (6)

$$g_{t}=tanh(V_{c}[h_{t-1},x_{t}]+a_{c})$$ (7)

$$c_{t}=f_{t}*c_{t-1}+i_{t}*g_{t}$$ (8)

$$o_{t}=\sigma (V_{o}[h_{t-1},x_{t}]+a_{o})$$ (9)

$$h_{t}=o_{t}*tanh(c_{t})$$ (10)

Where Eq 4 is the forget cell equation, Eq 5 is the input gate equation,

Eq 6 is the candidate cell state equation, Eq 7 is the cell state equation,

Eq 8 is the output gate equation, Eq 9 is the hidden state equation, and h_*t*−1_ is the previous hidden state, x_*t*_ is the input at time step *t*, σ is the sigmoid

activation function, tanh is the hyperbolic tangent activation function, V_*f*_, V_*i*_, V_*C*_, V_*o*_

are weight matrices and a_*f*_, a_*i*_, a_*C*_, a_*o*_ are bias vectors.

### Support vector regression algorithm

Two forms of support vector machines (SVMs) are Support Vector Regression (SVR) and Support Vector Classification (SVC), respectively utilized for regression analysis and classification [55]. Comparable to other regression models. SVR uses a margin of error (epsilon) to control the error level in the model, and a function called kernel is used to convert the input data into a higher dimensional space where the data is more separable. SVR aims to discover a hyperplane in the higher dimensional space to acquire the maximum margin of error while still obtaining the maximum amount of data. The prediction of new data points is made using a regression hyperplane [56]. The mathematical expressions for SVR [57] are described in Eqs (11–16):

1. The primal optimization problem:

**minimize**:

The objective function is composed of two parts:

$$\frac{1}{2}{\left\|w\right\|}^{2}+C\sum_{i=1}^{n}\left(\xi_{i}+{\xi_{i}}^{*}\right)$$ (11)

The first component is the regularization term $\frac{1}{2}{\left\|w\right\|}^{2}$, which minimises the weight vector w’s squared norm in order to penalize the model’s complexity. The penalty term, which is the product of the slack variables ξ_*i*_ and the product of ξ_i_* and a regularization parameter C. This variable regulates the trade-off between mistake minimization and margin maximization.

**subject to**:

$$y_{i}-w^{T}\emptyset (x_{i})-b\leq \in +\xi_{i}$$ (12)

$$w^{T}\emptyset (x_{i})+b-y_{i}\leq \in +{\xi_{i}}^{*}$$ (13)

$$\xi_{i}{\xi_{i}}^{*}\geq 0$$ (14)

These are the restrictions that govern the primary optimization problem. By using slack variables ξ_*i*_ to penalize violations, they guarantee that the model makes predictions within a margin of error ∈ of the actual target values additionally ξ_i_*.

2. The dual optimization problem:

**maximize**:

$$\sum_{i=1}^{n}\alpha_{i}^{*}-\sum_{i=1}^{n}a_{i}-\frac{1}{2}\sum_{i=1}^{n}\sum_{j=1}^{n}\left(a_{i}-a_{i}^{*}\right)(a_{j}-a_{j}^{*})\langle \emptyset (x_{j}),\emptyset (x_{j})\rangle$$ (15)

subject to:

$$\sum_{i=1}^{n}(a_{i}-a_{i}^{*})=0\;0\leq a_{i},a_{i}^{*}\leq C$$ (16)

With the goal of maximizing the margin between support vectors, the dual problem is constructed. In this case, *a*_*i*_ and $a_{i}^{*}$ are the Lagrange multipliers linked to the primal constraints, and 〈∅(*x*_*j*_),∅(*x*_*j*_)〉 denotes the inner product in the feature space caused by the kernel function.

## IV. Wind speed forecast model establishment

### Gharo wind turbine and data collection

Gharo is a small town in Pakistan’s Thatta district, Sindh province. The Gharo wind mast is located on the town’s outskirts. The meteorological mast has an NRG data collecting system installed. The atmospheric sensors’ specifications are displayed in Table 2. The site’s topographical coordinates are 24°35’48”N and 67°26’39”E. The wind speed data is collected at a time stamp of ten minutes for fifteen days.

**Table 2:** Atmospheric sensor’s parameters [48].

	Wind Speed Sensor	Temperature Sensor
Sensors	Cup type (M# 40)	6—ICT radiation plate (M # 110)
Operative assortment	190 m/s	4052.5°C
Correctness	±0.8%	±1.1°C
Temperature assortment	5560°C	4052.5°C
Distance constant	3.0 m	-
Display assortment	0120 Hz	02.5 V DC
Weight	0.2 kg	0.5 kg

### Data preprocessing

**Fig 3:**
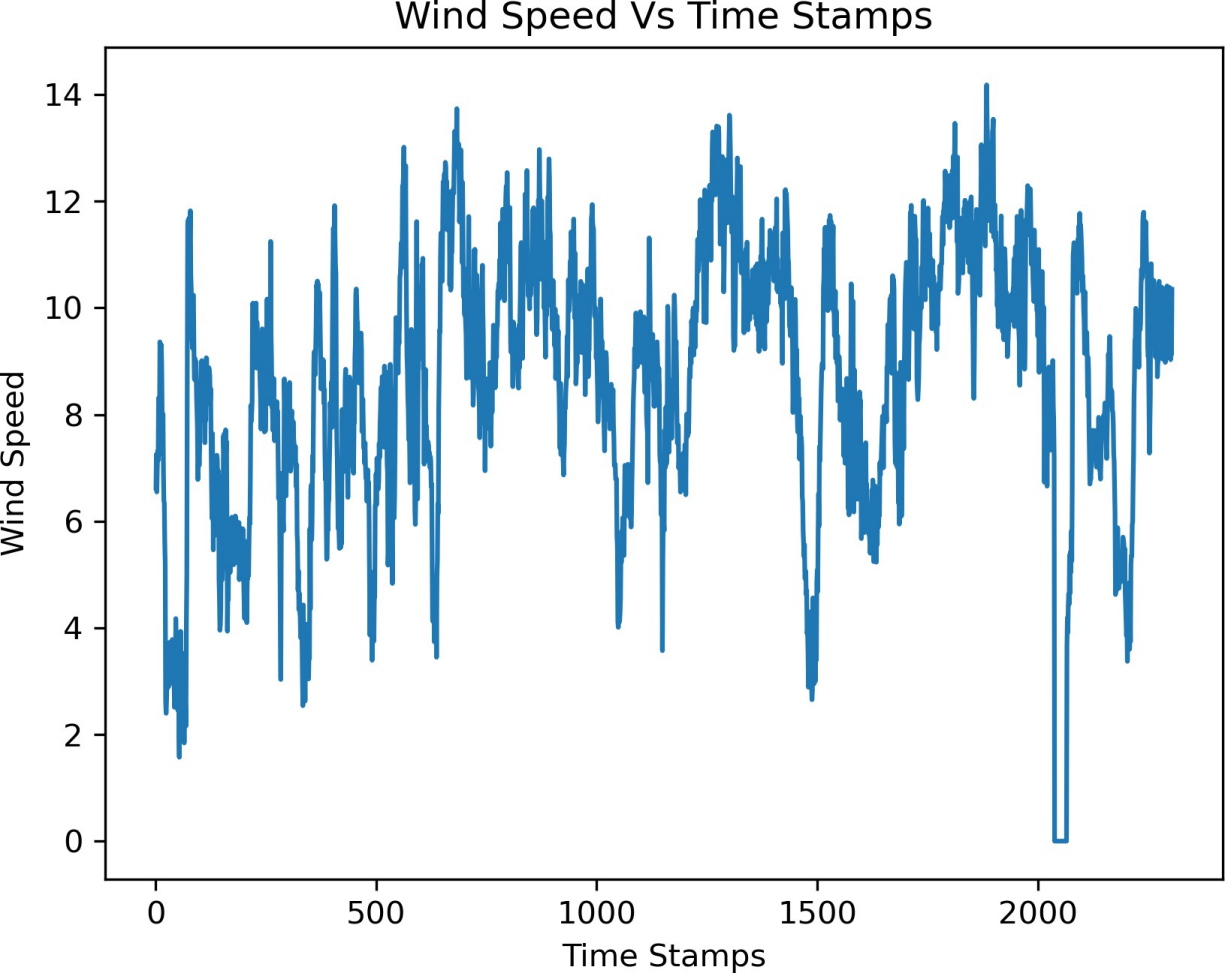
Wind speed plot.

The wind speed information comes from a Gharo wind turbine in Karachi, Pakistan’s city. The dataset spans September 15, 2016, to September 30, 2016, with a total of 2300 data samples, and the statistical summary of the dataset are shown in Table 3. As can be seen from the table, the experimental data fluctuates, ranging from 14.180 to 0.000, and the mean of the data set is 8.466088. Each data reading is taken at a timestamp of ten minutes. The plot of wind speed is shown in Fig 3. The data set is split into training, validation, and testing data for forecasting wind speed. The proposed model is trained on 1600 data samples, and verification and testing are done using 350 data samples. The platform used for wind speed prediction is Jupyter Notebook and programming language is Python 3.8.0 on HP Notebook laptop.

**Table 3:** Statistical summary of data source.

Abbreviation	Definition	Values
Count	The number of samples	2304
Mean	The average number of samples	8.466088
Std	Standard Deviation	2.836710
Min	Minimum Value	0.000
Max	Maximum Value	14.180

### Construction of the proposed model

**Fig 4:**
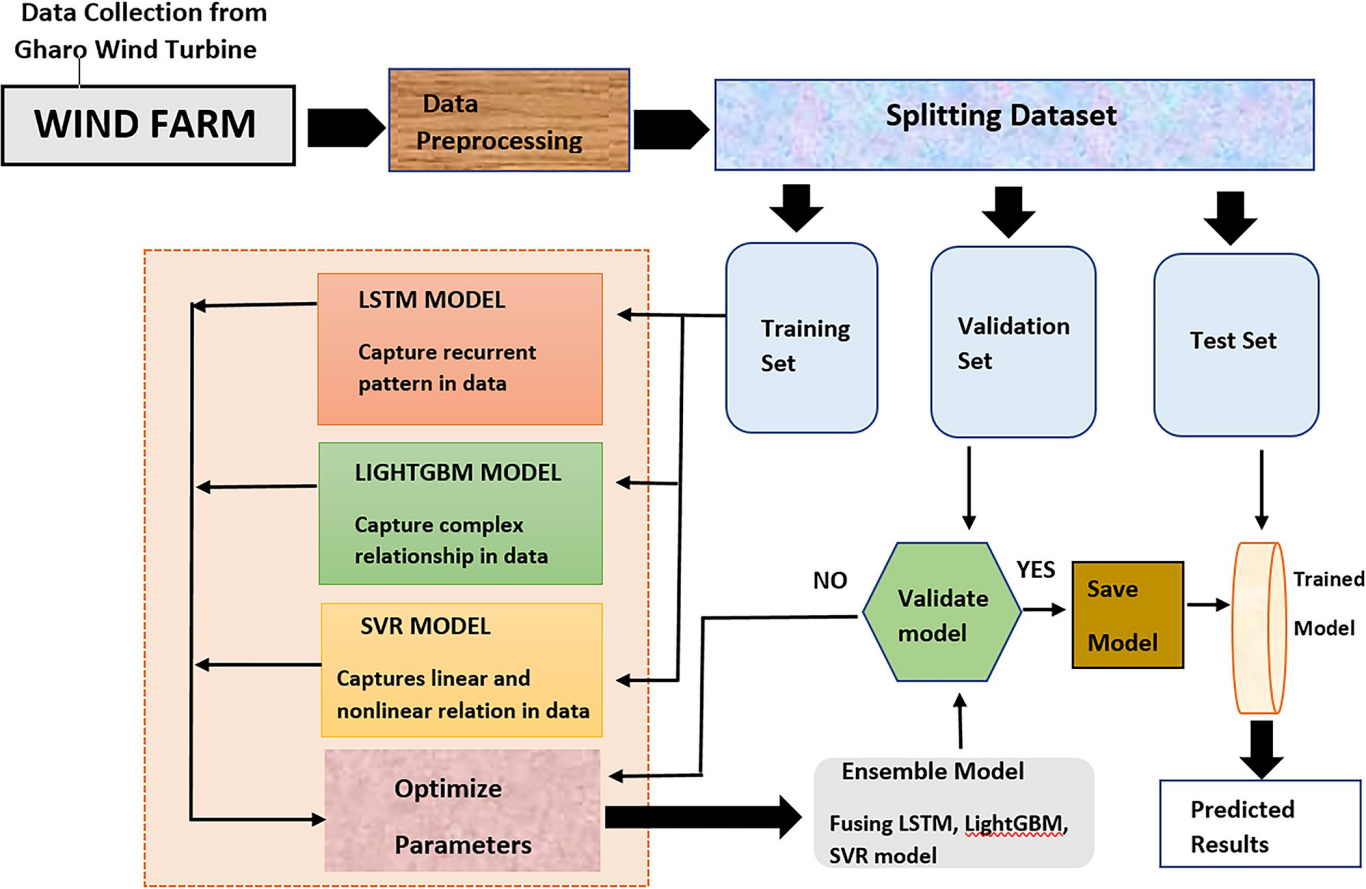
The architecture of proposed model.

Fig 4 describes the detail overview of an experimental setup of the proposed system. At first in the data is extracted from wind turbines, which is then preprocessed before being fed into our L-LG-S model. L-LG-S model is develop by combining LSTM, LightGBM and SVR using an ensemble method. The data is first preprocessed, and the LSTM, LightGBM, and SVR models are given a training dataset with 1600 samples. Sequential patterns are captured by the LSTM model; minute, hour, and day characteristics are captured by LightGBM; and both linear and nonlinear patterns are captured by SVR, which also creates hyperplanes to predict.

These models go under a hyperparameter tuning process using the Grid search method to achieve the optimal parameter value, as shown in Table 5. The ensemble technique is then utilized to fuse these models and validate the proposed model training process; the validation dataset of 350 samples is used to predict. Finally, the trained model is saved and deployed to test prediction after validation. As shown in Fig 4, the suggested model’s complexity and interpret ability are more tolerable, outperforming the benchmark models’ accuracy. The evaluation metrics of training, validation, and test prediction of the proposed model are given in Table 7.

### Evaluation indicator

The root mean square error (RMSE), mean squared error (MSE), and mean absolute error (MAE) are the most popular evaluation metrics in regression analysis [58, 59]. They are frequently employed in regression analyses to assess the prediction model’s performance. The difference between what was expected and what happened is less when the RMSE, MSE, and MAE are smaller. Table 4 contains a list of the RMSE, MSE, and MAE mathematical expressions. In these equations, n is the number of samples, *x*_*i*_ is the true value of the ith sample, and ${\hat{x}}_{i}$ is the predicted value of the ith sample.

**Table 4:** Mathematical expression of evaluation metrics.

Evaluation Metrics	Mathematical Expression
RSME	$\sqrt{\frac{1}{n}\sum_{i=1}^{n}{\left(x_{i}-{\hat{x}}_{i}\right)}^{2}}$
MSE	$\frac{1}{n}\sum_{i=1}^{n}{\left(x_{i}-{\hat{x}}_{i}\right)}^{2}$
MAE	$\frac{1}{n}\sum_{i=1}^{n}|x_{i}-{\hat{x}}_{i}|$

### Hyperparameter tuning

To improve the accuracy of predictions made by machine learning models, hyperparameter tuning is performed. The ideal value takes advantage of the benchmark models to operate more effectively inside the search domain and aids in avoiding underperformance due to incorrect parameters. For hyperparameter optimization in this paper, the grid search approach is used. This technique is frequently employed while optimizing hyperparameters. It offers a tailored search range and is simple to apply for. In this study, the hybrid models use default parameters while the individual test models are modified. Tables 5 and 6 show the search range and the best value for each individual benchmark model, respectively.

**Table 5:** Grid search range of parameters for benchmark models.

Models	Parameters	Grid search range of parameters
LSTM	Window size	6 to 24
	Layers	[16, 32, 64, 128]
Lazyprophet	Change points prior scale	[0.01, 0.1, 1.0]
	Seasonality prior scale	[0.01, 0.1, 1.0]
	Holidays prior scale	[0.01, 0.1, 1.0]
LightGBM	n estimaters	[100, 200, 300,. . ., 1000]
	learning rate	[0.001, 0.01, 0.1]
	max depth	[5, 10, 15, –1]
	num leaves	[20, 30, 40,. . ., 100]
	min child samples	[10, 20, 30,. . ., 100]
	subsample	[0.6, 0.7, 0.8,. . ., 1.0]
	colsample bytree	[0.6, 0.7, 0.8,. . ., 1.0]
RNN	Seq length	5 to 100,
	neurons units	16 to 256 units
	learning rate	0.0001 to 0.1
	epochs	50 to 500 epochs
SVR	C	[0.1, 1, 10, 100],
	Gamma epsilon	[0.001, 0.01, 0.1, 1], [0.01, 0.1, 0.5, 1]
CNN	learning rate	0.0001 to 0.1
	Kernel size	3 to 5
	no of filters	10–100
	Pooling Window Size	2 to 4

**Table 6:** Optimal parameter set for benchmark models.

Models	Parameters	Optimal value
LSTM	Window size	24
	Layers	50
	epochs	50
	batch size	64
	optimizer	Adam
Lazyprophet	Change points prior scale	0.01
	Seasonality prior scale	0.01
	Holidays prior scale	0.01
LightGBM	n estimaters	200
	learning rate	0.001
	max depth	-1
	num leaves	40
	min child samples	60
	subsample	0.7
	colsample bytree	0.9999
RNN	Seq length	24
	neurons units	64
	optimizer	Adam
	learning rate	0.1
	epochs	50
	batch size	32
	scaler	MinMaxScaler
SVR	C	100
	Gamma epsilon	0.1
		0.01
CNN	learning rate	0.01
	batch size	32
	no of epochs	50
	optimizer	Adam
	Kernel size	3
	activation	Relu
	no of filters	32
	Pooling Window Size	2

## V. Result and discussion

### Experimental results

**Fig 5:**
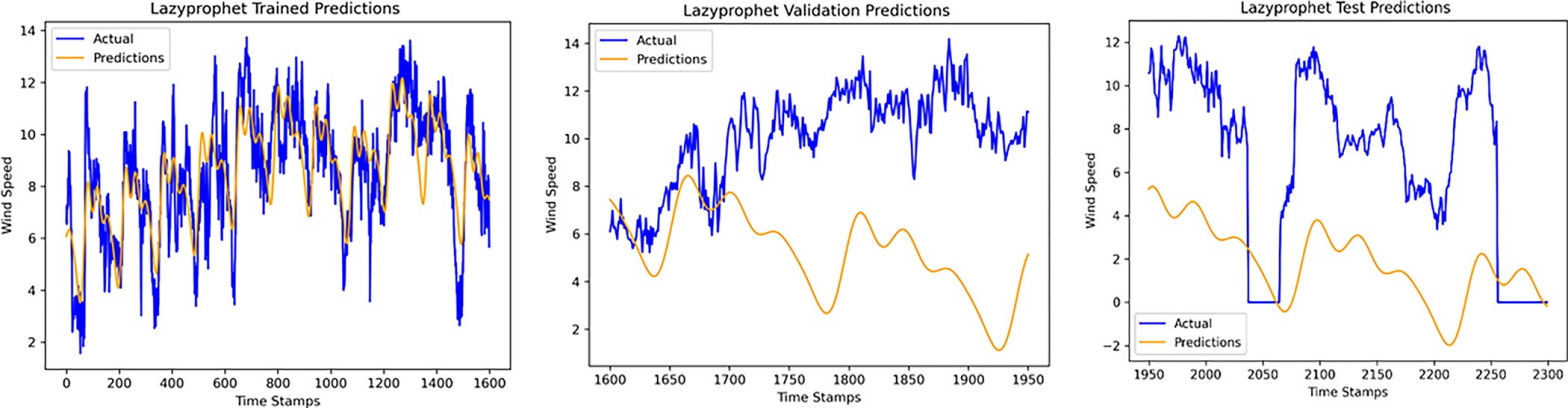
Lazyprophet model predictions.

**Fig 6:**
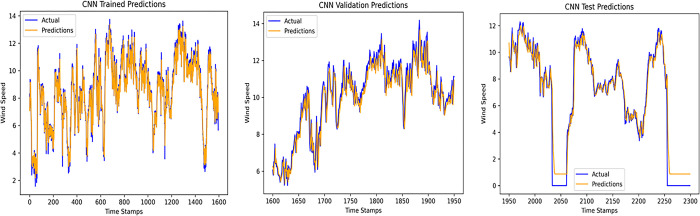
CNN model predictions.

**Fig 7:**
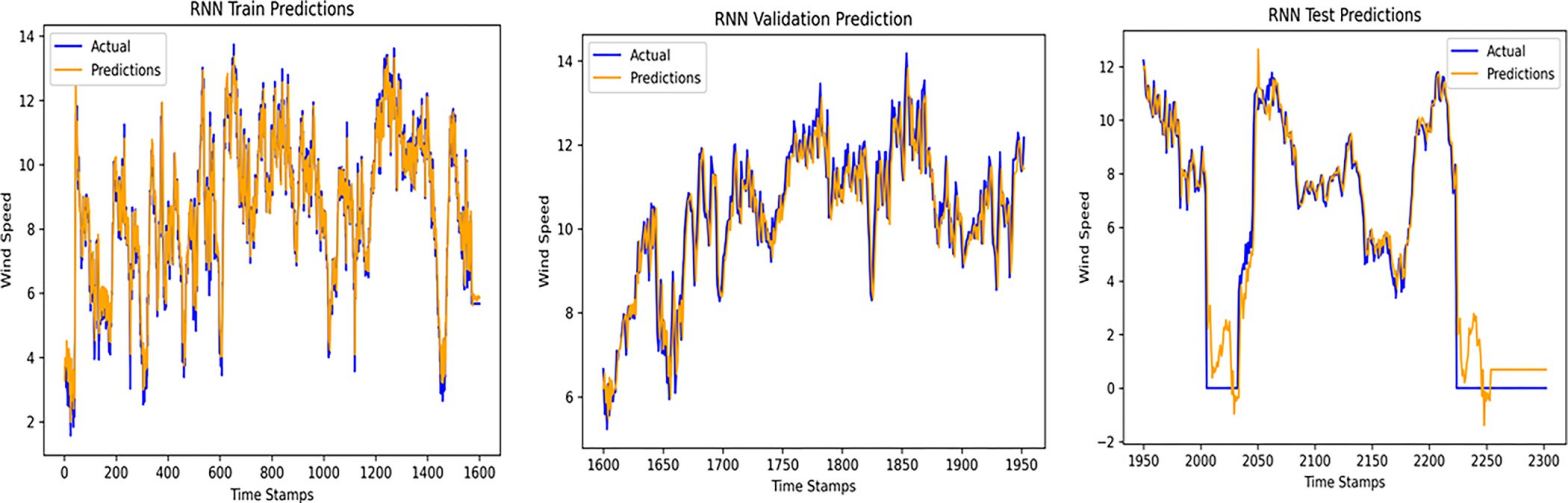
RNN model predictions.

**Fig 8:**
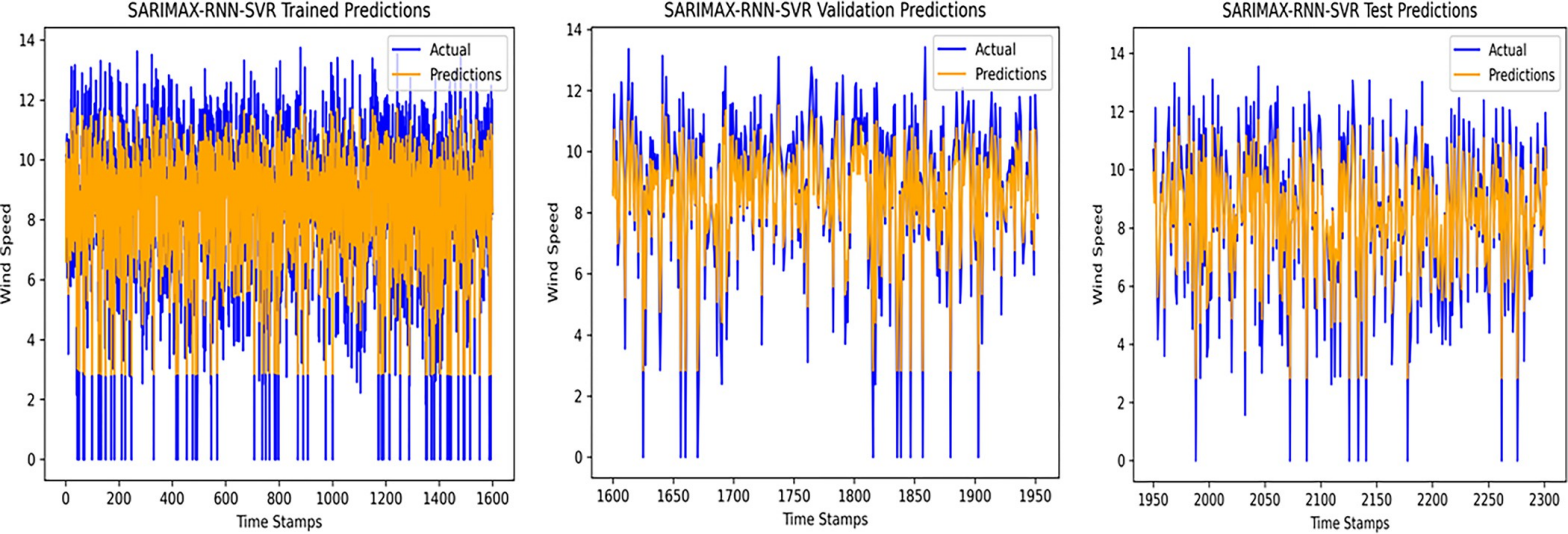
SARIMAX-RNN-SVR model predictions.

**Fig 9:**
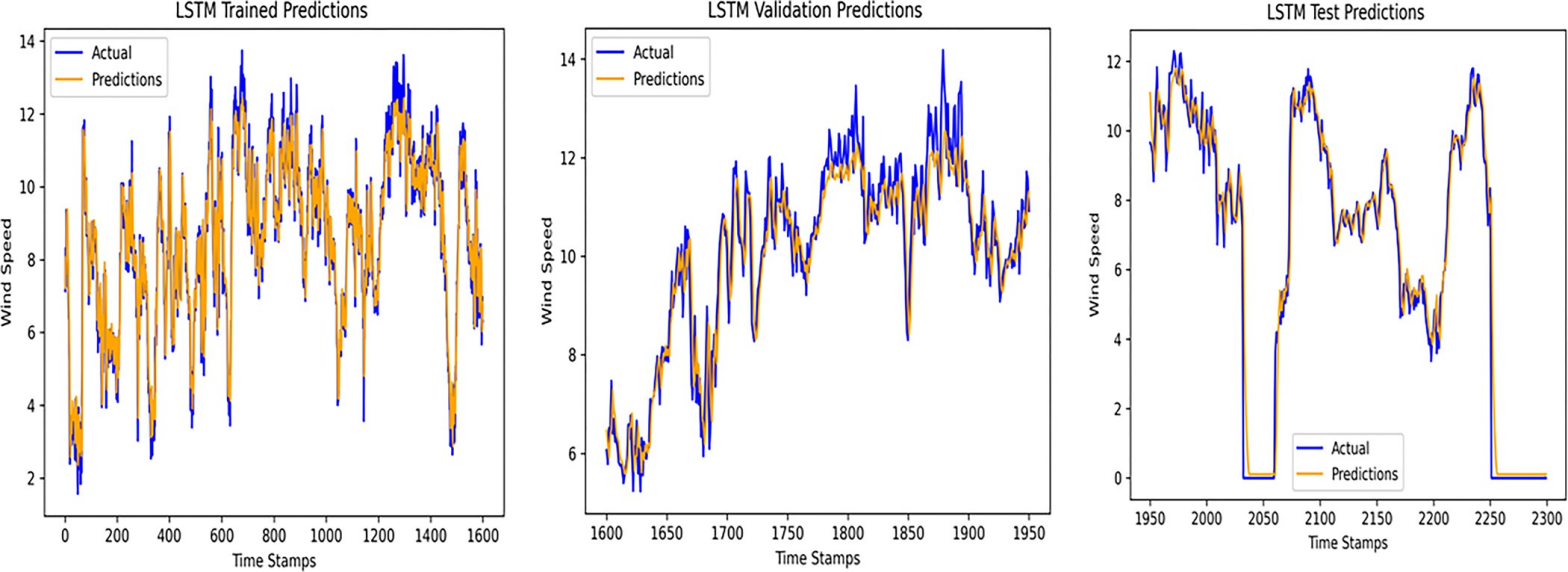
LSTM model predictions.

**Fig 10:**
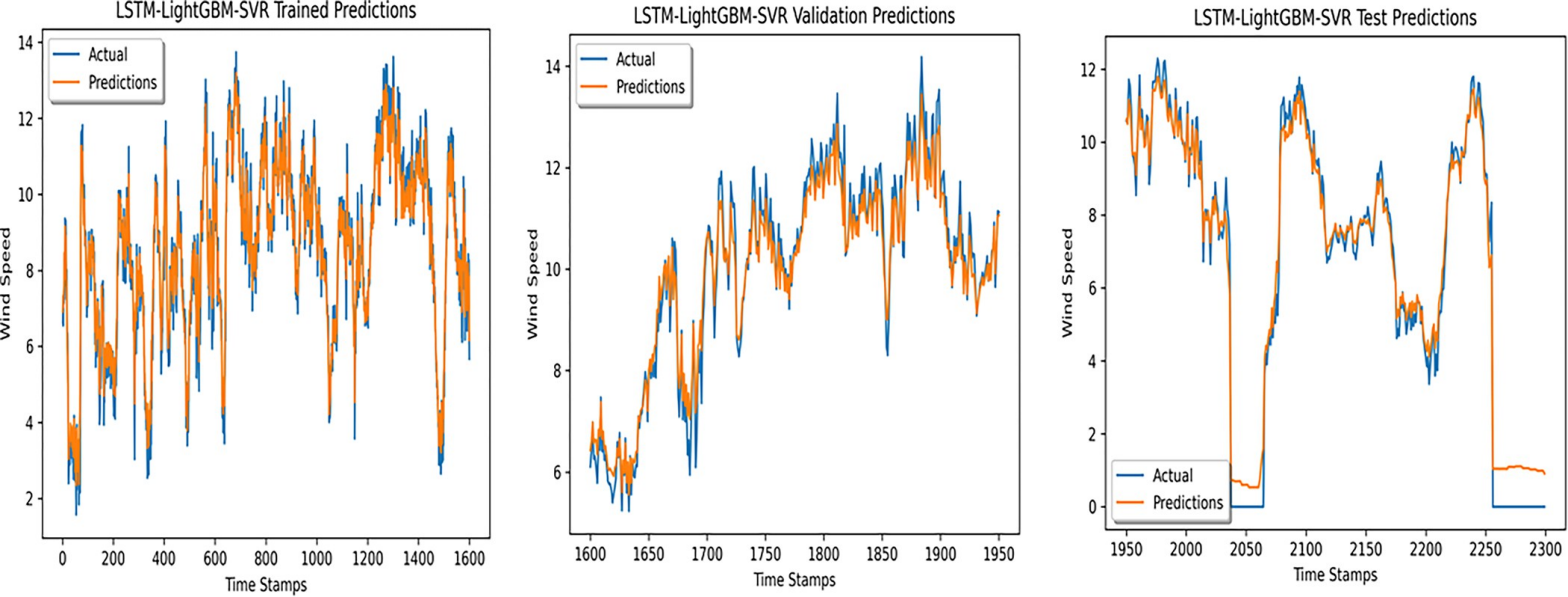
L-LG-S (Proposed) model predictions.

An accurate wind speed forecast is essential for efficient wind farm operation and dependable wind power generation. However, due to the stochastic and erratic character of wind speed, time series WSP is a challenging problem in forecasting. The short-term WSP is essential for managing wind turbines and extending their lifespan and power generation efficiency. This research employs the proposed model L-LG-S for short-term wind speed prediction, whose data source is mentioned in Section IV. In this paper, the proposed model is compared with popularly known state of the art prediction models available in literature, CNN, Lazyprophet, LSTM, RNN, and SARIMAX-RNN-SVR to determine its effectiveness. Figs 5–10 depicts the forecasting results of models. Among the benchmark models, LSTM network show promising results in wind speed prediction due to their ability to capture long-range dependencies. LazyProphet is a promising time series predictive model in the past few years, RNN, and CNN are representative deep neural network models with sound prediction effects for time series. SARIMAX-RNN-SVR is a hybrid model which has shown in commendable results in author’s previous work. These comparison models are more efficient and can verify how well the suggested model predicts the wind speed.

### Experimental analysis

The prediction performance of proposed and benchmark models are assessed using evaluation metrics, including RSME, MSE, and MAE. In Table 7, the evaluation of unique models shows that LSTM performs the best in training, validation, and test predictions, surpassing the other individual models. CNN achieves the second-best performance, though it outperforms LSTM in training, but in validation and test prediction, it stands after LSTM. The remaining models can be ranked in terms of their performance based on validation and test predictions as follows: SARIMAX-RNN-SVR, RNN, Lazyprophet. Although these models perform well in predicting wind speed. However, to capture wind speed’s stochastic nature and be more accurate in forecasting, this paper proposes a new hybrid model, L-LG-S, to enhance the accuracy and reliability of wind speed forecasts. This proposed model harnesses the strengths of LSTM, LightGBM, and SVR algorithms where the bold text indicates the predictions made by the model we have proposed. It is evident from Table 7 that the proposed model surpasses other state of the art models with train, validation, and test MSE, RSME, and MAE, 0.13, 0.36 and 0.28, 0.11, 0.34 and 0.27, 0.30, 0.55, and 0.43 respectively. The slightest errors in the predictions of the proposed model establish it as a reliable and accurate short-term wind speed prediction model.

**Table 7:** Evaluation metric of benchmark model and proposed model.

Evaluation Metrics			
Models	MSE	RSME	MAE
Trained Predictions			
Lazyprophet	2.13	1.46	1.17
RNN	1.22	1.11	0.87
LSTM	0.54	0.74	0.56
CNN	0.53	0.73	0.55
SARIMAX-RNN-SVR	0.92	0.96	0.74
Proposed Model	**0.13**	**0.36**	**0.28**
Validation Predictions			
Lazyprophet	31.30	5.59	4.83
RNN	1.18	1.39	1.00
LSTM	0.45	0.67	0.52
CNN	0.49	0.70	0.56
SARIMAX-RNN-SVR	0.86	0.93	0.73
Proposed Model	**0.11**	**0.34**	**0.27**
Test Predictions			
Lazyprophet	35.23	5.94	5.30
RNN	1.34	1.31	0.81
LSTM	0.86	0.92	0.51
CNN	0.95	0.97	0.63
SARIMAX-RNN-SVR	0.96	0.98	0.78
Proposed Model	**0.30**	**0.55**	**0.43**

## VI. Conclusion

Climate change and rising demand for electricity have led to the rapid development of clean energies like wind power in recent years. For certain power systems, wind power generation in particular becomes a major energy source. However, wind power generation forecasting and scheduling can be exceedingly challenging due to the unpredictable and irregular behavior of wind speed. This research presents the development and effectiveness of a novel hybrid model, L-LG-S, for wind speed prediction at a small wind farm located in Pakistan. In order to represent the stochastic nature of wind speed in the short-term horizon, the suggested model combines the strengths of LightGBM, SVR, and LSTM. An extensive evaluation of the proposed model’s performance is carried out utilizing statistical error indicators, namely MAE, RMSE, and MSE. The L-LG-S findings show that the train, validation, and test prediction errors for MSE, MAE, and RMSE are closer to zero at 0.13, 0.36, 0.28, 0.11, 0.34, and 0.27, 0.30, 0.55, and 0.43, respectively. To validate the suggested framework, comparisons are made using real wind speed plot, LSTM, CNN, SARIMAX-RNN-SVR, Lazyprophet, and RNN models. The simulation results demonstrated that the suggested model is suitable for short-term wind speed forecasting and outperforms legacy models.

## VII. Future work

This research work is focused on developing an accurate forecasting model to predict wind speed in short-term horizon and it has limitations for long-term predictions. The reduction in computational complexity and time will be the future work for this research work to become commercial system.
